# Food as Medicine Clinic: Early Results and Lessons Learned

**DOI:** 10.7759/cureus.31912

**Published:** 2022-11-26

**Authors:** David Hu, Anna Cherian, Kevin Chagin, Jennifer Bier, Douglas Einstadter, Douglas Gunzler, Alissa Glenn, Ellen McLaughlin, Karen Cook, James Misak, Shari D Bolen

**Affiliations:** 1 Medicine, Case Western Reserve University School of Medicine, Cleveland, USA; 2 Medicine, Northeast Ohio Medical University School of Medicine, Rootstown, USA; 3 Institute for Health Through Opportunity, Partnership, and Empowerment (HOPE) and Population Health Innovations Institute, The MetroHealth System, Cleveland, USA; 4 Food and Nutrition Services, The MetroHealth System, Cleveland, USA; 5 Medicine, Case Western Reserve University at The MetroHealth System, Cleveland, USA; 6 School of Urban Affairs, Cleveland State University and the Greater Cleveland Food Bank, Cleveland, USA; 7 Family Medicine, Case Western Reserve University at The MetroHealth System, Cleveland, USA

**Keywords:** chronic disease management, food and nutrition, implementation science, primary care, food insecurity

## Abstract

Introduction: Hospital-based food pantries are commonly used to address food insecurity. However, few studies have examined the impact of these food pantries on patients with chronic health conditions. In this study, we sought to assess the effect of a hospital-based food pantry clinic on self-reported dietary changes, health outcomes, and resource utilization.

Methods: This study included food insecure participants with suboptimally controlled congestive heart failure, hypertension, or diabetes who visited a Food as Medicine (FAM) clinic at an academic healthcare system between October 2018 and November 2019. The clinic provided a three-day supply of food for participants and their families up to two times per month for up to 12 months. Baseline, three-month, and six-month surveys were used to assess dietary behaviors, and electronic health record (EHR) data were used to assess health outcomes and utilization. Multivariable Poisson regression was used to explore variables associated with FAM clinic use.

Results: At three months, participants self-reported improved dietary behaviors, including increased consumption of fruits and vegetables as snacks and an increased variety of fruits and vegetables consumed. There were no statistically significant changes in clinical or healthcare utilization measures, despite small absolute improvements in systolic blood pressure (SBP), hospitalizations, and emergency department (ED) visits. There was a weak association between FAM clinic visit frequency and changes in dietary behaviors.

Conclusion: Among patients with chronic diseases, the use of the FAM clinic was associated with improved self-reported dietary behaviors and a nonsignificant improvement in health outcomes and resource utilization.

## Introduction

Recently, health systems have shown increased interest in addressing social determinants of health, which have been shown to affect health outcomes to a greater extent than clinical care alone [1,2]. One social determinant that has been of critical focus is food insecurity, since access to affordable healthy foods, such as fruits and vegetables, is necessary for diets that improve the health of people with chronic conditions [3]. Food insecurity, defined as limited or uncertain availability and access to nutritionally adequate and safe foods [4,5], is an economic and social condition that affected 10.5% of US households in 2019 [6,7]. Among adults, food insecurity is associated with an increased risk of developing chronic conditions such as diabetes, hypertension, and depression [8-11]. In persons with diabetes, food insecurity is associated with poorer blood glucose monitoring, increased emergency department (ED) visits for hypoglycemia, and cost-related medication non-adherence [12,13]. When compared to their food-secure counterparts, food-insecure people are also more likely to incur increased expenditures for hospitalizations and medications - a gap that is even wider in food-insecure persons with chronic conditions [14]. Given the potential for worse health outcomes and higher healthcare costs, there has been considerable interest in interventions that alleviate food insecurity and enhance access to healthy foods.

Healthcare professionals, public health advocates, and health systems have explored the use of different modalities of food assistance, such as fruit and vegetable voucher programs, medically tailored food delivery services, produce prescription programs, and food bank referrals, with mostly positive findings on behavior change [15]. Healthcare systems have also established hospital-based food pantries to improve accessibility and tailor food for a patient’s chronic conditions, with the hope that this tailored effort will have an impact on health outcomes and costs. However, few studies have examined the impact of hospital-based food pantries. These prior studies have described self-reported improved convenience, increased trust in food selection, and reduced stigma surrounding utilization when compared to the use of a community-based food bank [16,17]. However, to the best of our knowledge, no study has evaluated the effect of a hospital-based food pantry on changes in health outcome measures and self-reported dietary consumption among food-insecure patient populations living with chronic disease (e.g., hypertension, diabetes, heart failure). These high-risk patients may benefit the most from such an intervention.

Therefore, we decided to implement a hospital-based food pantry, named the Food as Medicine (FAM) clinic, and evaluate its effect on self-reported dietary consumption, health outcomes, and utilization. We hypothesized that addressing food insecurity for chronic disease patients would improve self-reported dietary behavior, improve health outcomes, and reduce healthcare utilization. To facilitate others implementing hospital-based food pantries, we also sought to identify factors associated with greater engagement with the FAM clinic and determine if greater engagement impacted health behaviors.

## Materials and methods

Study design

We conducted a pre-post study using prospective surveys and retrospective electronic health record (EHR) data to evaluate changes in dietary behaviors, program engagement, and health outcomes. Participants completed surveys at their initial visit and at visits three and six months later. We limited the study timeline to patients enrolled between October 2018 and November 2019 to allow for a minimum of three months of follow-up prior to the start of the COVID-19 pandemic in March 2020. The MetroHealth System IRB approved the study protocol IRB18-00640 on August 31, 2018.

Study population and setting

The FAM clinic is located adjacent to the main hospital of the MetroHealth System, a safety-net healthcare system in northeast Ohio. The MetroHealth System includes 3 hospitals and 20 outpatient clinics throughout the Cleveland Metropolitan Area. Patients eligible for inclusion included adults 18 years of age and older with a primary care provider in the MetroHealth System who had food insecurity based on answering yes to either of the two-item short forms of the United States Department of Agriculture Household Food Security survey [15] and a chronic health condition. Chronic health conditions included congestive heart failure with recent hospitalization, hypertension with the most recent blood pressure measurement greater than 150/100 mmHg, or diabetes with the most recent hemoglobin A1c (A1c) greater than 7%. We selected these conditions because increased consumption of fruits and vegetables may be associated with clinical improvement. There were no exclusion criteria.

Referrals to the FAM clinic came from a variety of sources, including inpatient and outpatient case managers, social workers, physicians, dietitians, and nursing staff. Patients could also self-refer, and the staff at the FAM clinic would determine their eligibility. The FAM clinic enrolled eligible patients on an ongoing basis. Patients remained enrolled for up to a year or until the patient stated that they did not have a need for ongoing food support. We referred individuals who did not meet inclusion criteria to the Greater Cleveland Food Bank’s program to connect people with community food resources.

Intervention

Referred individuals could visit the FAM clinic and receive up to a three-day supply of food for their entire household at each visit. Open hours were Wednesdays from 1 PM to 5 PM and Thursdays and Fridays from 9:30 AM to 1:30 PM. Starting in July 2019, we extended our open hours to Tuesday afternoons. A trained dietetic technician assisted patients in determining medically tailored food choices (such as low-salt options for patients with hypertension). A volunteer shopping assistant helped patients select individual food items, ensured the appropriate quantities for household size, and packed the groceries in reusable shopping bags. The FAM clinic offered additional nutrition information, recipes, and nutritional counseling at each visit, in addition to follow-up visit reminder cards and transportation/parking vouchers if necessary. Participants could visit the FAM clinic twice per month for up to 12 months.

Measures/outcomes

The primary study outcome was change in self-reported dietary behavior, defined as an improvement in fruit and vegetable intake. Secondary outcomes included other dietary behaviors such as reduction in fast food intake, the number of visits to the FAM clinic, changes in clinical health measurements (i.e. blood pressure, A1c, body mass index (BMI), and health care utilization [i.e., ED use and inpatient hospitalizations)]. We also identified potential barriers to FAM clinic use.

Data collection

At the initial visit, we collected demographic information [age, gender, race/ethnicity, education, transportation, referral type, and any use of government food assistance programs (e.g. Supplemental Nutrition Assistance Program)], and administered a survey to assess dietary and behavioral patterns based on a validated dietary checklist [16]. For returning patients, we repeated the survey at 3 and 6 months. We used the EHR to obtain information on health metrics (A1c, blood pressure, and BMI), utilization of the FAM pantry, and any hospitalizations or ED visits. The dietetic technician documented in the EHR at each FAM visit when the patient received food. Clinic staff reached out to patients who missed visits to identify possible reasons for missed visits. We reviewed these notes to assess potential barriers to program engagement.

Data analysis

We recorded patient characteristics from the initial survey and carried forward characteristics for the three- and six-month follow-up surveys. We report patient characteristics as counts (%) for categorical variables and means (standard deviation) for continuous variables.

To evaluate patient dietary changes between the initial survey and the three-month follow-up survey at the three-month visit, we used a non-parametric Wilcoxon sign test for paired results. We describe the dietary changes between the initial and six-month follow-up surveys in Table 1, but we did not conduct further quantitative analyses of differences as the sample size was too low to provide sufficient power to detect significant differences.

**Table 1:** Self-reported dietary changes from baseline to six months of FAM clinic use Numbers represent counts (%). ^a^p-value calculated using a paired nonparametric Wilcoxon test.

Dietary variables	6 months (n = 19)		
	Initial	Follow-up	p-value^a^
Fruits or vegetables as snacks?			
No	1 (5%)	0 (0%)	0.212
Sometimes	10 (53%)	9 (47%)	
Often	5 (26%)	6 (32%)	
Every day	3 (16%)	4 (21%)	
Missing	0	0	
Citrus fruit or juice in the past week?			
No	5 (26%)	6 (32%)	0.770
Yes	14 (74%)	13 (68%)	
Missing	0	0	
How much fruit daily?			
None	1 (5%)	1 (5%)	0.518
½ cup	4 (21%)	3 (16%)	
1 cup	6 (32%)	8 (42%)	
1½ cup	3 (16%)	2 (10%)	
2 cups	3 (16%)	4 (21%)	
2 ½ cups	1 (5%)	0 (0%)	
3+ cups	1 (5%)	1 (5%)	
Missing	0	1	
More than one kind of fruit daily?			
No	4 (21%)	2 (11%)	0.351
Sometimes	10 (53%)	13 (68%)	
Often	4 (21%)	3 (16%)	
Everyday	1 (5%)	1 (5%)	
Missing	0	1	
How many vegetables daily?			
None	0 (0%)	1 (5%)	0.265
½ cup	4 (21%)	5 (25%)	
1 cup	8 (42%)	5 (26%)	
1½ cup	2 (10%)	0 (0%)	
2 cups	3 (16%)	4 (21%)	
2½ cups	0 (0%)	2 (10%)	
3+ cups	2 (10%)	2 (10%)	
Missing	0	0	
More than one kind of vegetable daily?			
No	6 (32%)	1 (5%)	0.065
Sometimes	7 (37%)	11 (58%)	
Often	6 (21%)	5 (26%)	
Every day	2 (10%)	2 (11%)	
Missing	0	0	
Two or more vegetables at the main meal?			
No	4 (21%)	3 (16%)	0.308
Sometimes	10 (53%)	13 (68%)	
Often	3 (16%)	2 (11%)	
Every day	2 (10%)	1 (5%)	
Missing	0	7	
Fast food in the past 7 days?			
No	13 (68%)	17 (90%)	0.203
Sometimes	3 (16%)	2 (10%)	
Often	2 (11%)	0 (0%)	
Every day	1 (5%)	0 (0%)	
Missing	0	0	

A second analysis of dietary changes looked at the relationship between the three-month dietary change and engagement in the FAM program. We regressed the number of FAM clinic visits on the change between the initial and three-month surveys using a linear regression model. We controlled the number of FAM clinic visits for a time by calculating the ratio between the number of FAM visits and the length of time a participant engaged in the program; a larger ratio indicates that more visits per month occurred during the participant’s enrollment.

We measured changes within a participant’s clinical health measurements (i.e., A1c, BP, and BMI) and healthcare utilization between the patient’s initial FAM visit and their last visit to the clinic. For the initial measurement, we used the latest value recorded during the one-year period prior to the initial FAM visit. For the follow-up measurement, we used the latest value recorded during the one-year period after the last FAM visit. Our analysis excluded participants who did not have recorded vital signs or laboratory assessments within these time intervals. We measured hospitalizations and ED visits as the number of visits per month in the one-year pre and one-year post-periods. We utilized a non-parametric Wilcoxon Sign test to compare pre- and post-values.

We used Poisson regression to determine variables associated with FAM clinic use among enrolled patients. Patient-level variables included age, gender, race, ethnicity, the patient’s distance to the FAM clinic, transportation type (car, bus, or other), referral type (categorized as self-referred, inpatient referral, outpatient referral, or other), the Charlson Comorbidity Index [17], the area deprivation index [18], and the length of time the patient had been enrolled in the program. We selected these characteristics for the regression model because they could potentially affect changes to the FAM program and were accessible within the survey and EHR data. We utilized program enrollment length to control the association between the number of visits and the participant’s enrollment duration. The analysis used all follow-up time and did not exclude patients who may have been lost to follow-up prior to their three- and six-month visits. For all analyses, we used a p-value less than 0.05 to indicate statistical significance.

## Results

Study population characteristics

Overall, 79 participants had at least one FAM clinic visit and completed a baseline survey. At the time of enrollment, participants had a mean age of 57 years, were mostly female (68%), had a high school education or less (65%), and self-reported as either black (61%) or white (37%) (Table 2). Almost all participants (>86%) had hypertension and/or diabetes, while 23% had congestive heart failure. Many participants (63%) were obese (BMI) ≥ 30]. About half the participants either had an elevated A1c ≥ 7% (49%) or elevated systolic blood pressure (SBP) ≥ 130 mmHg (47%) using their most recent value prior to the visit. The inpatient service (42%) was the most common source for referrals, followed by self-referral (23%), and the outpatient internal medicine primary care clinic (19%). The most common transportation types were car (42%) and bus (34%), although many listed other forms of transportation. About half (48%) were currently receiving federal food assistance. The three- and six-month survey follow-up results were generally similar for patient demographics over time.

**Table 2:** Food as Medicine participant characteristics at baseline, three months, and six months Unless otherwise noted, numbers represent counts (%). Data are taken from the intake, 3-month, and 6-month surveys. ^a^SDOH: social determinants of health referrals occur when a care coordinator screens a patient for SDOH and identifies that they meet the eligibility criteria for the FAM program. ^b^“Other” includes referrals from OB/GYN or Dietitian. ^c^Participants could choose multiple answers for this question. A1C: hemoglobin A1c; BMI: body mass index; FAM: Food as Medicine; IM: internal medicine; SBP: systolic blood pressure; SD: standard deviation; SDOH: social determinants of health.

Characteristic	Baseline survey (n = 79)	3-month survey (n = 39)	6-month survey (n = 19)
Mean age, in years [SD]	57 [11.4]	58 [11.5]	58 [13.8]
Gender			
Female	54 (68%)	26 (67%)	12 (63%)
Ethnicity			
Hispanic	7 (9%)	3 (8%)	2 (10%)
Non-Hispanic	71 (90%)	36 (92%)	17 (90%)
Missing	1 (1%)	0 (0%)	0 (0%)
Race			
Black/African American	48 (61%)	23 (59%)	10 (53%)
White	29 (37%)	15 (38%)	8 (42%)
Missing	2 (2%)	1 (3%)	1 (5%)
Education			
Less than HS	23 (29%)	10 (26%)	6 (32%)
HS or GED	28 (36%)	14 (36%)	7 (37%)
Some college	18 (23%)	9 (23%)	3 (16%)
College degree or higher	9 (11%)	5 (13%)	3 (16%)
Missing	1 (1%)	1 (2%)	0 (0%)
Comorbidities			
Diabetes	65 (82%)	33 (85%)	17 (90%)
Hypertension	68 (86%)	34 (87%)	16 (84%)
Congestive heart failure	18 (23%)	8 (21%)	5 (26%)
A1c ≥ 7%	39 (49%)	19 (49%)	9 (47%)
Missing	20 (25%)	11 (28%)	4 (21%)
SBP ≥ 130 mmHg	37 (47%)	16 (41%)	7 (37%)
Missing	17 (22%)	10 (26%)	4 (21%)
BMI ≥ 30 kg/m^2^	50 (63%)	26 (67%)	9 (47%)
Missing	1 (1%)	0 (0%)	0 (0%)
Referral source			
Outpatient IM clinic	15 (19%)	8 (20%)	2 (11%)
Inpatient	33 (42%)	16 (41%)	13 (68%)
Self-referred	18 (23%)	12 (31%)	4 (21%)
SDOH^a^	9 (11%)	1 (3%)	0 (0%)
Other^b^	3 (4%)	2 (5%)	0 (0%)
Missing	1 (1%)	0 (0%)	0 (0%)
Transportation^c^			
Car	33 (42%)	15 (39%)	10 (53%)
Bus	27 (34%)	16 (41%)	6 (32%)
Other	32 (40%)	17 (44%)	5 (26%)
Federal Food Assistance			
I have heard of this program	14 (18%)	10 (26%)	6 (32%)
I have used this program	17 (21%)	4 (10%)	2 (10%)
I currently use this program	38 (48%)	21 (54%)	8 (42%)
Missing	10 (13%)	4 (10%)	3 (16%)

FAM clinic use

**Figure 1:**
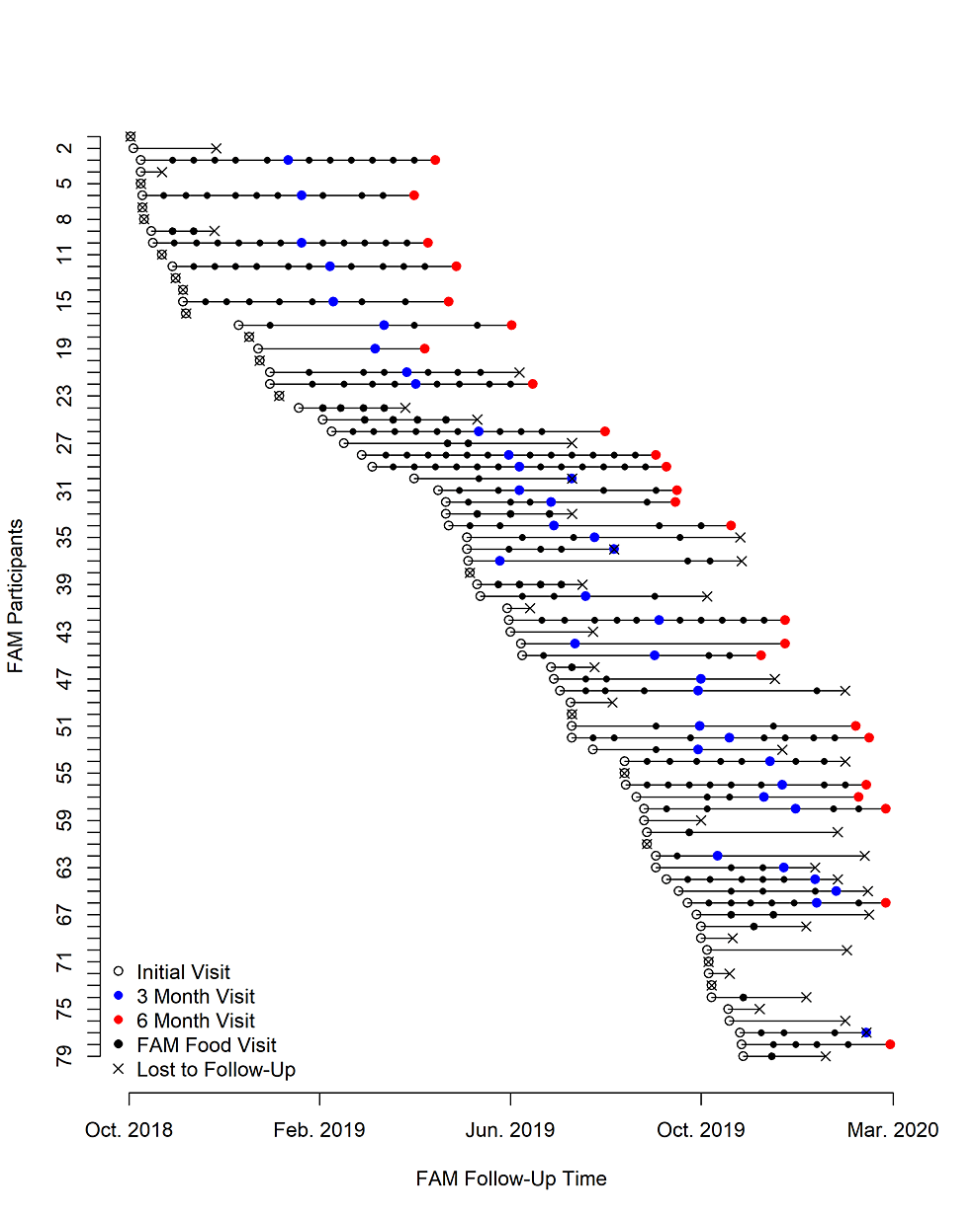
Time plot of number of visits to the Food as Medicine clinic (initial visit through March 2020 or loss to follow-up)

FAM clinic use was quite variable among participants (Figure 1). Of the 79 who started the program, only about 50% continued attending the FAM clinic for three months. By six months, this had decreased to about 25%. The median length of time between the initial and last FAM clinic visit for all 79 participants was 3.4 months [interquartile range (IQR): 0.6-8 months]. The demographics of the patients who followed up were similar to the baseline group. For participants with a three-month follow-up visit, the median number of food pantry visits was 3 (IQR 2-5), and for patients with a six-month follow-up visit, the median number of visits was 5 (IQR 2-7). We were unable to contact the majority of those who did not return to the FAM clinic. However, of the eight participants we reached, most (63%) cited competing demands or competing health issues as the reason for their inability to follow up. Other reasons for the lack of follow-up included forgetting, resolution of their food needs, and one death. After adjustment for demographics, transportation, distance from the clinic, comorbidity, area deprivation index, and length of time an individual was active in the program, white participants and those who self-referred made significantly more visits to the FAM clinic (Table 3).

**Table 3:** Factors associated with engagement in Food as Medicine clinic visits ^a^Length of time an individual participated in the program was used to control the association between number of visits within the period. CI: confidence interval; FAM: Food as Medicine; SDOH: social determinants of health.

Covariates	Expected no. of visits	95% CI	p-value
FAM follow-up length^a^	1.2122	(1.1837, 1.2541)	<0.0001
Age	1.0058	(0.9918, 1.0106)	0.8057
Gender			
Female	Ref	Ref	Ref
Male	0.9886	(0.7923, 1.2727)	0.9685
Ethnicity			
Hispanic	Ref	Ref	Ref
Non-Hispanic	0.6524	(0.5308, 1.2520)	0.3293
Race			
Black/African American	Ref	Ref	Ref
White	1.4893	(1.1652, 1.9000)	0.0014
Distance from the clinic (miles)	1.0253	(0.9844, 1.0648)	0.2121
Transportation			
Car	0.7925	(0.5794, 1.0843)	0.1456
Bus	1.1005	(0.8044, 1.5040)	0.5479
Other	0.8459	(0.6333, 1.1273)	0.2548
Referral type			
Inpatient	Ref	Ref	Ref
Internal medicine	1.3795	(0.9754, 1.9489)	0.0682
Other	1.6295	(0.8981, 2.8258)	0.0934
SDOH	1.4348	(0.8490, 2.3774)	0.1678
Self-referral	1.5451	(1.0946, 2.1859)	0.0136
Charlson Comorbidity Index	0.9807	(0.9385, 1.0238)	0.3792
Area Deprivation Index	0.9976	(0.9909, 1.0041)	0.4675

Dietary changes associated with FAM clinic use over time

**Figure 2:**
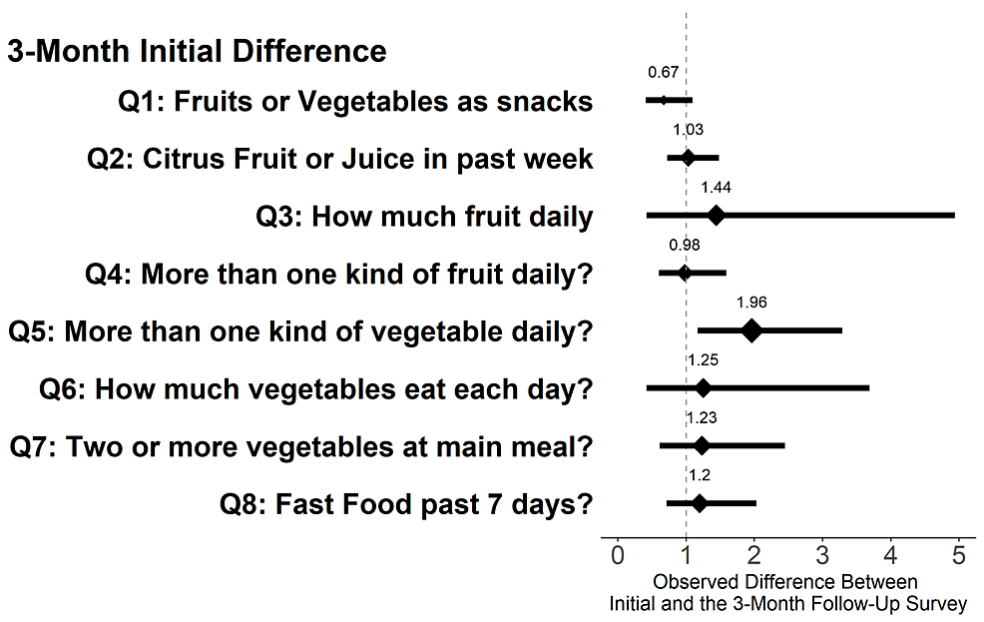
Analysis between high Food as Medicine clinic utilization and changes within the dietary and behavioral survey (initial visit to three-months) Note: Utilization of the Food as Medicine clinic is calculated as a ratio over the length of time the individual participated within the program. A higher number indicates more visits within a time frame than a smaller number.

Compared to the baseline, participants completing the three-month food survey (n = 39) reported eating significantly more fruits and vegetables as snacks, eating more than one type of vegetable daily, and eating less fast food within the last week (Table 4). At six months, survey respondents (n = 19) reported similar results to those at three months, though analyses were limited by the small sample size (Table 1). In a separate analysis, after adjusting for time in the program, we found no association between the number of FAM clinic visits and eating fruits or vegetables as snacks, total fruits, and vegetables eaten in a day, or fast food consumption. However, greater FAM clinic use was positively associated with self-reported eating of more than one type of vegetable daily (Figure 2).

**Table 4:** Self-reported dietary changes from baseline to 3 months of Food as Medicine clinic use ^a^p-value based on a paired nonparametric Wilcoxon test. *Means significant findings at p<0.05.

Dietary variables	3 months (n = 39)		
	Initial	Follow-up	p-value^a^
Fruits or vegetables as snacks?			
No	1 (3%)	0 (0%)	0.018*
Sometimes	19 (49%)	14 (37%)	
Often	10 (26%)	11 (29%)	
Every day	9 (23%)	12 (34%)	
Missing	0	1	
More than one kind of vegetable daily?			
No	9 (23%)	4 (10%)	0.029*
Sometimes	16 (41%)	16 (42%)	
Often	7 (18%)	11 (29%)	
Every day	7 (18%)	7 (18%)	
Missing	0	1	
Citrus fruit or juice in past week?			
No	11 (28%)	11 (29%)	0.790
Yes	28 (72%)	27 (71%)	
Missing	0	1	
How much fruit daily?			
None	4 (10%)	1 (3%)	0.868
½ cup	9 (23%)	10 (26%)	
1 cup	10 (26%)	13 (34%)	
1 ½ cup	4 (10%)	6 (16%)	
2 cups	7 (18%)	6 (16%)	
2 ½ cups	1 (3%)	1 (3%)	
3+ cups	4 (10%)	1 (3%)	
Missing	0	1	
More than one kind of fruit daily?			
No	9 (23%)	9 (21%)	0.239
Sometimes	20 (51%)	19 (50%)	
Often	8 (21%)	8 (21%)	
Everyday	2 (5%)	3 (8%)	
Missing	0	1	
How many vegetables daily?			
None	2 (5%)	1 (3%)	0.082
½ cup	9 (23%)	9 (24%)	
1 cup	14 (36%)	11 (29%)	
1½ cup	4 (10%)	3 (8%)	
2 cups	6 (15%)	7 (18%)	
2 ½ cups	0 (0%)	2 (5%)	
3+ cups	4 (10%)	5 (13%)	
Missing	0	1	
Two or more vegetables at the main meal?			
No	10 (26%)	4 (11%)	0.098
Sometimes	11 (44%)	21 (55%)	
Often	7 (18%)	7 (18%)	
Every day	5 (13%)	6 (16%)	
Missing	0	2	
Fast food in the past 7 days?			
No	24 (61%)	29 (76%)	0.033*
Sometimes	11 (28%)	9 (24%)	
Often	3 (8%)	0 (0%)	

FAM clinic effects on health outcomes and utilization

Only 74, 59, and 40 participants had a BMI, systolic blood pressure, or A1c measured, respectively, at baseline and after their last follow-up FAM clinic visit. Of these, 60, 36, and 27 participants had elevated values at baseline (BMI > 25 kg/m2, SBP ≥ 130, or A1c ≥ 7). All 79 participants had information on hospitalizations and ED visits for their initial and follow-up measurements. We found no statistically significant change in any measure over time, though there was a small decrease in systolic blood pressure, hospitalizations, and ED visits (Table 5).

**Table 5:** Median change in health outcomes and hospital utilization For the initial measurement, we used the latest value recorded during the one-year period prior to the initial FAM visit. For the follow-up measurement, we used the latest value recorded during the one-year period after the last FAM visit. ^a^p-value based on a nonparametric paired Wilcoxon test. A1C: hemoglobin A1c; BMI: body mass index; BP: blood pressure; IQR: interquartile range.

Measure	n	Median change (IQR)	p value^a^
BMI	74	0 (–5.2, 6.1)	0.741
BMI ≥ 25 kg/m^2^	60	0 (–5.4, 5.8)	0.888
Systolic BP	59	0 (–8, 15)	0.615
Systolic BP ≥ 130 mmHg	36	–0.5 (–20.8, 1)	0.119
A1c	40	0 (–1.4, 0.8)	0.374
A1c > 7%	27	0 (–1.4, 0.9)	0.348
Hospitalizations per month	79	–0.2 (–0.9, 0.5)	0.070
Emergency department visits per month	79	–0.1 (–0.7, 0.1)	0.078

## Discussion

Healthcare providers are becoming increasingly active in efforts to alleviate food insecurity and increase access to health-supportive foods. Recent research describes a variety of models employed to achieve these goals, including referrals to community-based food pantries [19-22], prescriptions/vouchers to farmers' markets [23-26], prescriptions/vouchers to retail food outlets [27,28], delivery of food bank-sourced produce to clinical sites [29], and food prescriptions to pantries located at the site of clinical care [30-32]. Many of these interventions have been associated with an increase in fruit and vegetable consumption and/or a decrease in food insecurity [19-22,24,26,27,29,33]. However, relatively few studies have evaluated the effect of food insecurity interventions on health outcomes. The exceptions involve two pilot interventions for patients with diabetes: one that provided diabetes-appropriate food boxes for pickup at neighborhood food pantries [20] and another that provided access to fresh produce in the clinical setting [29]. The only food as medicine research that, to the best of our knowledge, evaluates healthcare utilization is the very promising but small body of research on medically tailored meals for high-risk patients [14,34,35].

Furthermore, few studies have evaluated hospital-based food pantries, which are on the rise. Hospital-based food pantries have the potential to improve health since they can serve to enhance convenience by coordinating health and social services within one location, tailoring meals to specific health conditions [32], and allowing patients to re-engage in healthcare services as appropriate. Only two studies have described implementation processes and reported positive social and behavioral impacts of hospital-based food pantries on selected patient populations. For example, Greenthal et al. found that patients utilizing a hospital-based food pantry at Boston Medical Center had high satisfaction with the health condition-appropriate foods available, expressed comfort and trust with the staff and food environment, felt less stigma in the hospital pantry setting, and found the pantry convenient to access [32]. They also reported an increase in fruit and vegetable intake associated with the use of the pantry. Hickey et al. hypothesized that a clinic-based food pantry for pediatric patients would improve preventive healthcare service completion but failed to corroborate this finding among a 12- to 24-month-old patient population [31]. They did, however, find that the three-day emergency supply of food provided to food-insecure patients and their families resulted in increased trust in the clinic and improved personal circumstances, including flexibility to re-allocate limited resources to other family needs.

Our study adds to the existing literature by evaluating the impact of a hospital-based food pantry on health outcomes and healthcare utilization, describing patient engagement in a hospital-based food pantry focused on high-risk populations, and exploring the impact of patient engagement on self-reported dietary behaviors. Lessons learned from our FAM clinic could be used by others planning similar programs within their health systems. Our findings of improved self-reported dietary behavior, including fruit and vegetable consumption, are consistent with the existing food as medicine literature, and the observation that nearly half of our participants were current users of the SNAP program confirms that food insecurity remains prevalent in populations despite receiving federal food assistance [36]. Further, while we found no significant change in the short term, there was a suggestion of improvement in healthcare utilization and a slight decrease in systolic blood pressure among FAM participants. Additional studies with additional health outcome measures (such as lipids), larger sample sizes, and/or measurements over a longer period of time (at least 12 months for health care utilization metrics) will be necessary to explore these findings on health outcomes and utilization. Lastly, we found that self-referred and white patients visited the FAM clinic more frequently. While self-referred patients have been shown to be more likely to adhere to treatments when compared to those receiving outpatient referrals, likely due to increased motivation [37], racial and ethnic differences in FAM utilization may be related to additional barriers associated with structural racism or social drivers of health [38].

This study has several limitations. First, our sample size was relatively small due to challenges in recruiting participants to a new program, and we had less follow-up time than planned due to the onset of the COVID-19 pandemic. This limited our power to detect potential changes in health outcomes and utilization. Second, we did not have a comparison group for our findings. While we could have developed a matched cohort for the health outcomes and utilization, we did not observe significant changes in these outcomes, thus rendering a matched cohort less relevant. Finally, many study participants had missing clinical and laboratory data, which could have affected our findings related to health outcomes. Despite these limitations, the lessons learned around engagement and the promising initial findings should allow others to learn from our work as they develop programs to identify and address food insecurity. This is particularly relevant as the current Biden-Harris administration announced more than $8 billion in new commitments as part of a call to action for the White House Conference on Hunger, Nutrition, and Health in September 2022 [39]. One of the five pillars highlighted in the Department of Health and Human Services in their report on current federal nutrition support initiatives focused on ensuring our healthcare system addresses the nutrition-related needs of all people [40] and will be a continued focus in the new national strategic plan to address nutrition insecurity [39].

## Conclusions

In summary, the use of the FAM clinic by high-risk patients with chronic disease was associated with improved self-reported dietary health behaviors and potential improvements in health outcomes and resource utilization. Future research should focus on ways to enhance participants’ use of hospital-based food as medicine interventions over time and evaluate the effectiveness of these interventions on both short- and long-term health outcomes and healthcare utilization compared to other food as medicine interventions, such as those that require a referral to community food pantries, farmers' markets, or retail outlets for filling food prescriptions.
